# Three-Dimensional Sulfated Bacterial Cellulose/Gelatin Composite Scaffolds for Culturing Hepatocytes

**DOI:** 10.34133/cbsystems.0021

**Published:** 2023-04-12

**Authors:** Xinmeng Li, Weixiao Ding, Shujun Wang, Luyu Yang, Qingqing Yu, Changji Xiao, Guangbo Chen, Lei Zhang, Shanyue Guan, Dongping Sun

**Affiliations:** ^1^ Institute of Chemicobiology and Functional Materials, School of Chemistry and Chemical Engineering, Nanjing University of Science and Technology, 200 Xiao Ling Wei, Nanjing 210094, China.; ^2^ Department of Blood Transfusion, Jinling Hospital, Nanjing University School of Medicine, Nanjing 210002, China.; ^3^ Obstetrics and Gynaecology Department, Peking University First Hospital, Peking University, Beijing 100034, China.; ^4^ Center for Advancing Electronics Dresden (Cfaed) and Faculty of Chemistry and Food Chemistry, Technische Universität Dresden, 01062 Dresden, Germany.; ^5^ Key Laboratory of Photochemical Conversion and Optoelectronic Materials, Technical Institute of Physics and Chemistry, Chinese Academy of Sciences, Beijing 100190, China.

## Abstract

The liver is the hub of human metabolism and involves many diseases. To better work on the mechanism and treatment of liver diseases, it is of particular interest to design 3-dimensional scaffolds suitable for culturing hepatocytes in vitro to simulate their metabolic and regenerative abilities. In this study, sulfated bacterial cellulose (SBC) was prepared as the building block of cell scaffolds, motivated by the anionic nature and 3-dimensional structure of hepatic extracellular matrix, and its reaction condition for sulfate esterification was optimized by changing the reaction time. The analysis and study of the microscopic morphology, structure, and cytocompatibility of SBCs showed that they possess good biocompatibility and meet the requirements for tissue engineering. Next, SBC was mixed with gelatin for composite scaffolds (SBC/Gel) for culturing hepatocytes by homogenization and freeze-drying methods, whose physical properties such as pore size, porosity, and compression properties were compared with gelatin (Gel) scaffolds as the control group, and the cytological activity and hemocompatibility of the composite scaffolds were investigated. The results showed that the SBC/Gel composite has better porosity and compression properties, as well as good cytocompatibility and hemocompatibility, and could be applied to 3-dimensional culture of hepatocytes for drug screening or liver tissue engineering.

## Introduction

As a key hub for many physiological processes, liver plays an important role in a variety of bodily functions, such as the detoxification of systemic and portal blood and the secretion of many proteins and bile components [1]. Meanwhile, as the center of metabolism in the body, liver is susceptible to damage and failure, leading to cirrhosis, fatty liver, liver fibrosis, and liver cancer. Culturing hepatocytes, the major parenchymal liver cell, especially in a 3-dimensional (3D) form, mimics both the metabolic and regenerative functions of the liver and therefore provides indispensable roles in studying liver diseases [2]. Hepatocytes cultured 2-dimensionally (2D) differs substantial from the microenvironment in which cells are grown in vivo and some key cellular phenotypes are lost. For example, in the 2D cell culture of primary human hepatocytes, the expression of many liver-specific functional genes and proteins is rapidly lost after passaging [3]. 3D cell culture, on the other hand, allows cells to adhere, proliferate, and migrate within materials of 3D structures, which more accurately simulate cellular conditions in the human microenvironment than in 2D cell culture. In addition to being a model to better understand common diseases such as hepatocellular carcinoma, a 3D culture model of hepatocytes has a wide range of applications in toxicology screening and development, regenerative medicine, and tissue engineering [4,5].

Seed cells, scaffolds, and growth factors are the 3 main elements of tissue engineering. Scaffolds are to create the conditions for cell growth and improve cell adhesion, proliferation, cytokine secretion, and cell-to-cell interactions to grow into tissue organs with specific morphology [6]. To meet the requirements for 3D culture, scaffolds should possess good biocompatibility [7], in vivo degradation rate, pore size, mechanical properties, antimicrobial capacity, and hemocompatibility [8], which are to mimic the structure of extracellular matrix (ECM). As the major components of ECM, glycosaminoglycans (GAGs) usually have a large number of negatively charged groups, such as carboxyl and sulfate groups, and bind positively charged amino acids through electrostatic interactions [9]. GAG chains involve in important biological processes such as cell proliferation, differentiation, coagulation, infection, inflammation, and tumor metastasis by binding to cytokines, growth factors, and enzymes in living organisms [10].

Among them, heparan sulfate and chondroitin sulfates are important components in liver ECM, which modulate the function of cytokines through specific interactions and modulation with cytokines. Therefore, many studies have been conducted by establishing heparin- or heparan-like structures in the scaffolds. Zhang’s team [11] immobilized heparin and vancomycin on 3D printed Ti6Al4V scaffolds and showed that the heparinized scaffolds had anticoagulant and anti-infective abilities. Matsuno and coworkers developed a fibrin glue/fibronectin/heparin based delivery system for the controlled release of bone morphogenetic protein 2 and demonstrated that the addition of heparin could achieve a slow release of bone morphogenetic protein 2 without significantly affecting the structure and stiffness of fibroblasts [12].

We therefore hypothesized that sulfation of polysaccharides such as cellulose would mimic the natural structure of liver ECM and favors hepatocytes culturing. Bacterial cellulose (BC) is a natural nanostructured polymeric material produced mainly by microorganisms. BC not only has excellent properties such as high specific surface area, high water holding capacity, and high crystallinity but also has good biosynthetic modifiability and bioactivity and other excellent properties such excellent mechanical properties and has been shown to be a good scaffold material for tissue engineering [13]. Bhattacharya’s team prepared materials to support the growth of human hepatocytes (HepG2 and HepaRG) on the basis of BC and nanocellulose [14]. The sulfation of BC would help bind growth factors and increase the anticoagulant properties of the material, while maintaining the in situ 3D structure of BC, and therefore would facilitate cell growth as scaffolds [15]. The sulfate modification of BC is a chemical method to make the sulfate group replace the hydrogen on the hydroxyl group of BC molecule, which can make the polysaccharide have the biological activity similar to GAG. Commonly used reagents for sulfate esterification are ClSO_3_H chlorosulfonic acid [16], NH_2_SO_3_H, SO_3_-Py [17], etc. The degree of substitution of sulfate esterification is related to the reaction conditions. Within a certain extent, the degree of substitution of cellulose sulfation can be increased by increasing the reaction temperature, increasing the reaction time, and changing the ratio of sulfate esterifying agent to cellulose [18].

In this study, sulfated bacterial celluloses (SBCs) modified with sulfate were used as nanomaterials to composite with gelatin to improve the poor mechanical properties and rapid degradation rate of gelatin (Gel) scaffolds. The incorporation of gelatin improves the biocompatibility of BC, while SBC would prevent gelatin from rapid degradation, lack of strength and toughness, susceptibility to collapse of the scaffold material, and its tendency to swell when exposed to water [19].The SBCs not only improve the mechanical properties of the composite but also mimic the biological activity of the natural sulfated GAG, resulting in a 3D cell culture scaffold with good cytocompatibility and hemocompatibility and an effective heparin-like delivery system. We investigate whether this composite pair can be used for hepatocyte culture in vitro, opening up new ideas for implantation into tissue engineering research.

## Materials and Methods

### Materials

Sulfamic acid (99.5%) and calcium chloride anhydrous (96%) were purchased from Aladdin Reagent Co (China, Beijing). Urea, sodium hydroxide (NaOH, 96%) and gelatin (pH 5.0 to 7.0, ≥99.5) was purchased from Sinopharm Chemical Reagent Co. Ltd (Shanghai, China). N,N-dimethylformamide (>99.9%) was purchased from Shanghai Macklin Biochemical Co., Ltd (Shanghai, China). Cell Counting Kit-8 (CCK-8) was purchased from Beyotime Institute of Biotechnology (Shanghai, China). All the chemicals were used without further purification if no further specification was given. The microorganism used in this study was *Konagataeibacter nataicola* RZS01 [20], which was initially isolated from natural sources and stocked in our laboratory [21].

### Synthesis of SBC

As reported previously, self-assembled BC fibers were collected as pellicles on the surface of culture medium after static fermentation of *Komagataeibacter nataicola* RZS01, boiled with 0.3% NaOH at 80 °C for 6 h, and washed with distilled water until pH became neutral [22]. After high-pressure homogenization, BC membranes became slurries and were freeze-dried as BC powders, kept in dry and cold places for further use.

[Fig F1]After dissolving 2.5 g of sulfamic acid into 50 ml of N,N-dimethylformamide, 2 g of dried BC powder was added to the solution and dispersed by vigorous magnetic stirring. The mixture was heated in a metal bath at 50 °C for 2, 4, 8, or 12 h to prepare SBCs with different degrees of sulfate esterification [18], as indicated in Fig. 2. The reaction was stopped by adding a quantity of deionized water (DI water). The suspension was centrifuged at 10,000 rpm for 5 min and washed 3 times with DI water. After adjusting the pH of the suspension to 7.0 with 1 M NaOH solution, the obtained SBC suspension was dialyzed for 3 d against DI water and homogeneously dispersed using a homogenizer. The content of SBC suspension was adjusted to 6 g/ml.

**Fig. 1. F1:**
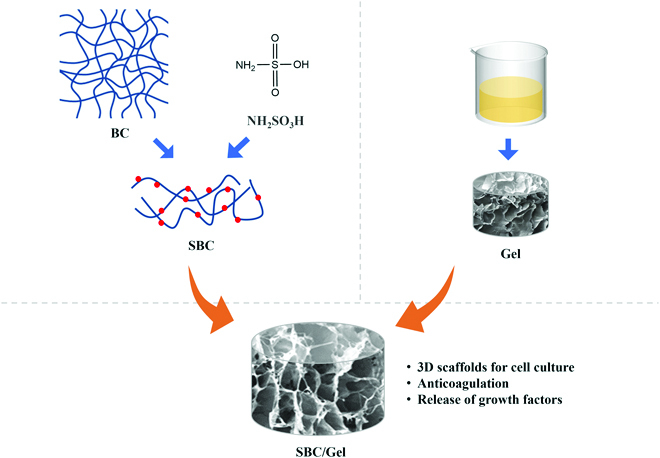
Schematic illustration of preparing SBC/Gel scaffold for 3D culture of hepatocytes.

**Fig. 2.**
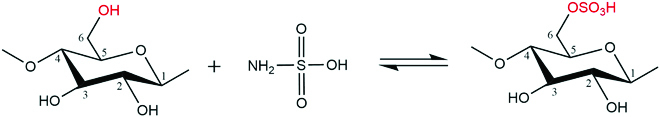
Strategy of introducing sulfate groups at C-6 positions of BC.

### Synthesis of gelatin and SBC/gelatin scaffolds

Gelatin pellets (0.1 g) were mixed with 10 ml of DI water or 10 ml of SBC suspension and gently heated to 37 °C in a metal bath to dissolve. The gelatin solution was pipetted into 96-well plates and prefrozen at −80 °C for 24 h. The material was then dried in a vacuum lyophilizer for 3 d for sponge-like porous gelatin scaffolds. The sole gelatin and the mixture of SBC and gelatin scaffold were denoted as Gel and SBC/Gel, respectively.

### Characterizations of materials

The content of sulfur in SBC samples was obtained by analyzing SBC samples with x-ray photoelectron spectrometry (XPS). The substitution degree of sulfate esterification was calculated according to the following equation [23]:

$${\text{DS}}_{\text{sul}}=\frac{S\%\times 162.1}{3027-102.1\times S\%}$$ (1)

Where DS_sul_ represents the degree of sulfation and S% represents the atomic percentage of S in the SBC.

The chemical structure of the samples was analyzed by Fourier transform infrared (FT-IR) spectroscopy (PerkinElmer Nicolet IS-20) with a scanning range of 500 to 4,000 cm^–1^ and a resolution of 4 cm^–1^.

The microstructure of the sample was observed by scanning electron microscopy (SEM) (JSM-IT500HR) after spraying a thin layer of gold on the surface of the sample to increase the electrical conductivity. The crystal structure of the samples was analyzed by a Bruker D8 Advance x-ray diffractometer (Cu Kα, λ = 1.5418 Å) at a voltage of 40 kV and a current of 35 mA in the range 5° < 2*θ* < 80°.

The zeta potential of BC and SBC suspensions at 6 mg/ml (pH 7) were measured using a Zetasizer Nano ZS (Malvern Instruments Ltd., Worcestershire, United Kingdom).

The degradability of the material was studied by in vitro degradation tests, where samples were completely immersed in phosphate-buffered saline (PBS) (pH 7.4) until equilibrium was reached. The samples were then placed in a shaker and incubated at 50 rpm and 37 °C for a predetermined period. Samples were removed at regular intervals and photographed. The volume of remaining material was used to reflect the degradability of the material.

The weighing method was utilized for measuring the porosity of the scaffolds. The dry weight of the lyophilized sample was weighed as *W_d_*. The material fully absorbed with anhydrous ethanol was weighed as *W_w_* after removing excess liquid with filter paper. After placing the soaked sample in a 25-ml measuring cylinder, more alcohol was added until the volume reached 10 ml. The volume of added alcohol was *V*_a_. The porosity of the sample was calculated by the following equation (Eq. 2):

$$P\left(\%\right)=\frac{\left(\mathrm{Ww}-\mathrm{Wd}\right)/\rho e}{10-\mathrm{Va}}$$ (2)

where ρ_e_ is the density of anhydrous ethanol.

To measure the mechanical properties of lyophilized scaffolds, the gelatin or SBC/gelatin samples were freeze-dried using 48-well plates to obtain scaffolds of approximately 10 mm in diameter and 10 mm in height. The compression stress–stain curves were recorded by a universal testing machine (TY8000-A500 N, China) at a stretching speed of 2 mm/min. The compression mechanical parameters were the average value of at least 3 parallel trials.

### Cell culture, proliferation, and cytotoxicity

Human fetal hepatocytes L02 cells (American Type Culture Collection, USA) were cultured in high-glucose Dulbecco’s modified Eagle medium (Gibco, USA) containing 10% fetal bovine serum (Gibco, USA), 100 units/ml of penicillin and 100 μg/ml of streptomycin (Beyotime, China) in a humidified cell incubator containing 5% CO_2_ at 37 °C.

For the studies of cell growth on scaffolds, the samples in the 96-well plates were sterilized by an ultraviolet lamp in the biosafety cabinet. After trypsinizing the L02 cells, 1 × 10^5^ ml^−1^ of cell suspensions were added directly into the sponge-like scaffold until it fully absorbs the cell culture medium. Two hours later, more freshly prepared cell culture medium was added into the culture plates to maintain the cell culture. After different durations of culture, CCK-8 assay (Solarbio, China) was performed to evaluate the cytotoxicity and cellular proliferation of the scaffolds according to the manufacturer’s protocol with slight modifications. CCK-8 reaction solution was added to each well of material and mixed well, where culture medium only was used as the blank group. After incubation in a cell culture incubator (37 °C, 5% CO_2_) for 3 h, 100 μl of the reaction solution was aspirated from each well, and the absorbance was measured using an enzyme marker (450 nm) with a reference wavelength of 630 nm. The growth curve of L02 cells was plotted from the optical density (OD) value.

To evaluate the effect of scaffolds on cell growth, after hepatocytes were cultured on the scaffold for 1 d, the cells were fixed with 4% paraformaldehyde (Soalrbio, China), washed with PBS 3 times, incubated with 0.1% Triton X-100 (Alfa Aesar, USA) in PBS for 10 min, and further stained with phalloidin-tetramethylrhodamine conjugate (C4233, APExBIO, China) and Hoechst 33342 (B8040, Solarbio, China) according to the protocols from the manufacturers. After the treatment, the cells were visualized via a confocal laser scanning microscope (FV3000, Olympus, Japan). The excitation wavelength for Hoechst 33342 was 405 nm, and the corresponding emission filter was 440 to 500 nm. The excitation wavelength for tetramethylrhodamine-labeled samples was 543 nm, and the corresponding emission filter was 565 to 650 nm.

### Anticoagulation property

The anticoagulant properties of Gel and SBC/Gel were analyzed using the activated partial thromboplastin time (APTT) by an automated coagulation analyzer CA-50 (Sysmex Co., Kobe, Japan). Healthy fresh human blood was collected intravenously in blood collection tubes and added with acid-citrate dextrose maintenance solution (containing 1.33 g of sodium citrate, 0.47 g of citrate, and 3 g of glucose per 100 ml) at a volume ratio of anticoagulant to blood of 1:4. By centrifuging the anticoagulated whole blood at 3,800 rpm for 10 min, removing the supernatant, and repeating the procedure one more time, platelet-poor plasma (PPP) was prepared. In all blood tests, blood sample from the same donor was used. For each test, 1 mg of scaffold material was incubated with 100 μl of plasma for 5 min at 37 °C before 50 μl of prewarmed APTT reagent (Siemens, Germany) was added. After incubating the mixture at 37 °C for 3 min, 50 μl of prewarmed CaCl_2_ solution (25 mmol·l^–1^) was added, mixed, and immediately placed in a coagulometer for testing. The images of the PPP were taken both before and after reaction.

## Results and Discussion

### The effect of reaction time on the degree of sulfate substitution in SBCs

**Fig. 3.**
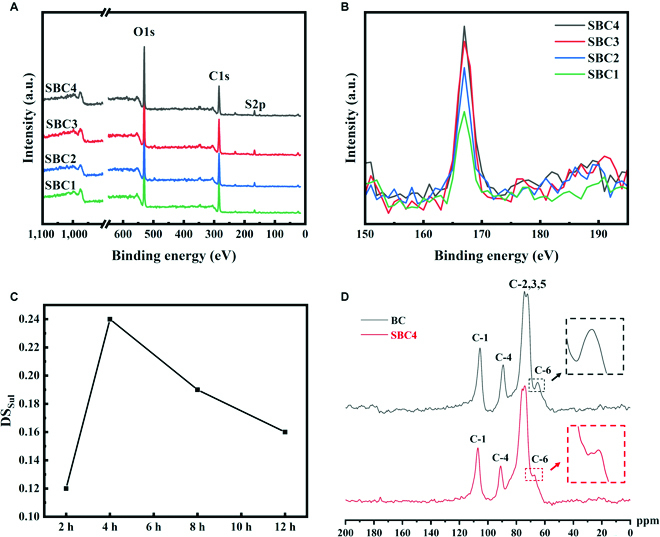
The substitution degree of sulfate groups in BC was related to reaction time. (A) Full XPS spectra of SBCs show existence of sulfur after reaction in all treatments. (B) Specifically, S2p spectra of SBCs indicate increased degrees of substitution (DS_sul_) from SBC1 to SBC4. (C) SBC1, SBC2, SBC3, and SBC4 refer to BC samples after reactions for 2, 12, 8, and 4 h, with DS_sul_ of 0.12, 0.16, 0.19, and 0.24, respectively. (D) 13C ssNMR spectrum of BC and SBC4 (DS_sul_ = 0.24). a.u., arbitrary units.

The anhydroglucose unit (AGU) refers to a single sugar molecule in a polymer. Each AGU is reduced to its functional groups, 3 hydroxyl groups per AGU. We prepared SBCs with different degrees of substitution by controlling the reaction time at the same content of sulfate ester (NH_2_SO_3_H: AGU = 2:1) and reaction temperature (50 °C). After reaction from 2 to 12 h, 4 groups of suspensions were freeze-dried to obtain sponge-like materials. We compared the as-obtained SBC1 to SBC4 and compared their degrees of sulfate substitution by x-ray photoelectron spectroscopy (Fig. 3). In Fig. 3A, despite the peaks indicating the electron binding energies of C 1s and O 1s at 284.8 and 531 eV, respectively, the new peak at 167 eV is attributed to the that of S 2p in all SBCs [24]. The result confirms that all SBCs contain sulfur in the form of S^6+^, identifying the presence of the sulfate group. We named the 4 SBCs as SBC1, SBC2, SBC3, and SBC4 in the order of increasing degree of substitution, as suggested by the intensity of the S 2p peak in Fig. 3B and the atomic percentage calculated accordingly (Fig. 3D).

Interestingly, the sulfate degree of substitution (DS_sul_), as calculated using Eq. 1, did not increase linearly with the extension of reaction time from 2 to 12 h but reached its maximum value at a reaction time of 4 h (Fig. 3C). Among them, SBC4 possesses the largest DS_sul_ of 0.24, while SBC1, SBC2, and SBC3 contain DS_sul_ of 0.12, 0.16, and 0.19, respectively. It is possible that as the time increases (2 to 4 h), the BC and sulfate reagents were mixed more thoroughly and the forward reaction dominates (Fig. 2). The weight contents of sulfur (S), carbon (C), and oxygen (O) elements in various SBC samples were measured by XPS, and the data are shown in Table. However, with further extension of the reaction time (8 to 12 h), as BC dehydrates in an acidic environment, as confirmed by the decreasing O content (from 46.2% to 39.7%), it would lead to the decrease of DS_sul_. The 13C NMR spectra of BC and SBC4 (DS = 0.24) were collected (Fig. 3D) to confirm the sulfation substitution reaction. The chemical shifts of C-6 are located at 65.03 ppm. The peaks of C-2, C-3, and C-5 locate from 72.31 to 77.34 ppm. After sulfation, the C-6 peak splits into two, one of which shifts downfield from 65.03 to 66.62 ppm, which verifies the substitution at the C-6 position [17]. Additionally, no shift was found in the peaks representing other hydroxyl groups. These findings together showed that the C-6 hydroxyl group of BC was partially substituted while the C-2 and C-3 hydroxyl groups were not substituted [16].

**Table.** The reaction conditions and atomic ratio of C, O, and S in resulting SBCs were summarized, where the DS_sul_ was calculated from the atomic ration of sulfur in each treatment

Samples	Conditions	C%	O%	S%	DS_sul_
SBC1	50 °C/2 h	51.7	46.2	2.1	0.12
SBC2	50 °C/12 h	57.7	39.7	2.7	0.16
SBC3	50 °C/8 h	52.6	44.2	3.2	0.19
SBC4	50 °C/4 h	50.1	46.0	3.9	0.24

**Fig. 4.**
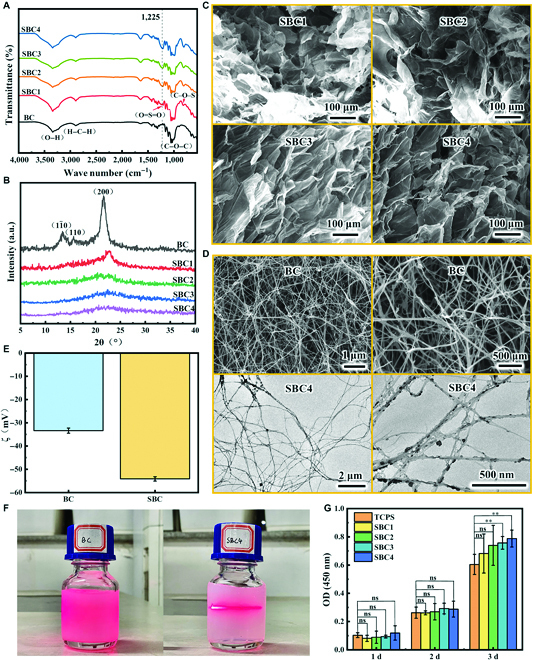
(A) FT-IR spectra and (B) x-ray diffraction spectra of BC and SBCs. (C) SEM images of SBCs, (D) SEM images of BC, and transmission electron microscopy image of SBC4 (DS_sul_ = 0.24). (E) Zeta potentials of BC and SBC4 at (dispersed in water, pH 7). (F) Optical images of BC and SBC4 dispersions scattered by a red laser beam, where BC was preprocessed by a high-speed shear homogenizer but did not show Tyndall phenomenon, while the SBC dispersion showed typical Tyndall phenomenon. (G) CCK-8 assay of L02 cells cultured on SBCs for different durations of time. **P < 0.01; ns, not significant.

The FT-IR spectra of BC and SBCs have a broad band at 3,400 cm^–1^ corresponding to the stretching vibration of the hydroxyl group (–OH) on BC (Fig. 4A). In addition, the bands at 2,920, 1,080, and 890 cm^–1^ correspond to the stretching vibrations of the C–H bond, C–O stretching vibrations, and C–H deformation vibrations [25], respectively, verifying the presence of polysaccharides in BC. In the FT-IR spectra of SBCs, 2 new bands appeared at 1,225 and 819 cm^–1^, corresponding to the O=S=O asymmetric stretching vibration and the C–O–S stretching vibration [24], proving the successful sulfate esterification reaction on BC. With increasing DS_sul_, the 2 new bands are also significantly enhanced, indicating the replacement of hydroxyl groups in BC that were substituted by the sulfate groups to an increased extent. The results correspond with the structural and morphological changes previously observed by x-ray diffraction (Fig. 4B), SEM (Fig. 4C), and transmission electron microscopy (Fig. 4D), indicating that the modification disrupts inter- and intramolecular interactions within BC fibers.

Generally, when zeta potential is greater than 30 mV or smaller than −30 mV, the dispersion is deemed stable. SBC dispersion at 6 mg/ml had a more negative zeta potential (−54.13 mV) (Fig. 4E) compared to the BC dispersion of the same solid concentration (−33.37 mV). The results were confirmed by the Tyndall effect (Fig. 4F), which indicates that the SBC solution is a well-stabilized colloidal solution. The increase in stability is mainly due to the esterification of the hydroxyl groups on the BC surface. On the one hand, the introduction of negatively charged sulfate groups (–OSO_3_^−^) onto the BC surface, and on the other hand, the sulfate esterification reaction disrupts the intra- and intermolecular interactions within the BC fiber, resulting in nanocolloidal particles with highly negative surface charge densities and a more stable colloidal solution of SBC due to electrostatic interactions.

Gelatin is obtained by hydrolysis of collagen and has been known to be noncytotoxic, so cytotoxicity of SBC is an important indicator of the compatibility of SBC/Gel scaffold. We compared the cytocompatibility of SBCs to polystyrene cell culture (TCP) plates by CCK-8 assay on L02 cells (Fig. 4G), of which the absorbance (OD) values reflects number of L02 cells. After cell culture for 24 h (day 1), the cell densities on SBCs were higher than TCP but increased and surpassed the 2D culture group as a control. It is possible that the number of viable cells on SBCs was lower because the cells did not adhere well to the 3D material in the beginning. However, the 3D scaffold provided a better growth environment for the cells, and they proliferated faster later. Specifically, the scaffold has larger surface area and interpenetrated pores that facilitate not only the migration of cells but also the entry and exit of waste and nutrients. In contrast, the TCP would gradually restrict cell growth space and the cell proliferation would lag behind due to the contact inhibition. In addition, among the 4 SBCs, those of higher degree of substitution slightly enhanced cell proliferation [26], probably because negative electrical properties facilitate cell adhesion and growth compared to neutral surfaces, and larger scaffold pore size makes the material more conducive to cell migration and nutrient flow. Above all, the growth rates of the 4 SBC groups were above 100%, indicating that the SBC materials of various substitution degrees were noncytotoxic and had good in vitro biocompatibility.

### Phyiscochemical characterizations of porous SBC/Gel composite scaffolds with different degrees of substitution

**Fig. 5.**
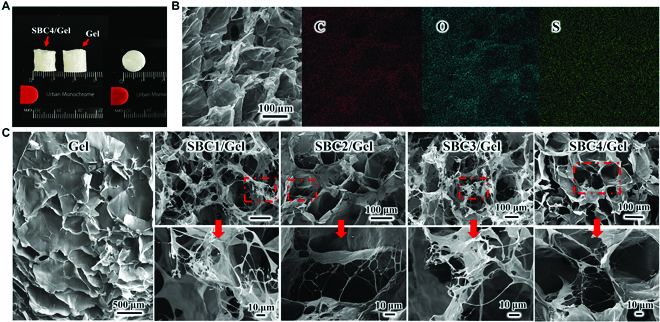
(A) Macroscopic morphology of the bottom and sides of the material after freeze drying. (B) Mapping image of the SBC1/Gel and (C) SEM images of Gel and SBC/Gels.

Figure 5A illustrates the macroscopic morphology of the SBC4/Gel composite aerogel after freeze-drying into 1 cm × 1 cm 3D porous scaffolds. Compared to sole gelatin (Gel) scaffold, one may find the composite has a certain degree of shrinkage. It may be that the SBCs contain a large number of hydroxyl and sulfate groups, which enhance the interactions between SBC and gelatin, causing a certain degree of shrinkage of the pore wall of the gelatin material. The homogeneous distribution of S elements, suggested by the elemental mapping image in Fig. 5B, indicates that SBC is uniformly dispersed in the gelatin, while the uniform distribution of other 2 elements, carbon and oxygen, also proves that SBC/Gel is a homogeneous material.

The microscopic images of SBC/Gel composites also showed highly porous structures. By SEM, we compared the morphologies of gelatin and SBC/Gel with different degrees of substitution (Fig. 5C), which showed that SBC/Gel materials have pores of approximately 200 to 300 μm in diameter, smaller than gelatin, but larger than the previous SBCs (Fig. 5C). The pore size of SBC/Gel scaffold gradually decreases and becomes more regular with the increase of substitution degree, possibly due to the strengthened interactions between SBC and gelatin, resulting in shrinkage of the pore size of the material. In addition, Fig. 5C shows a microscopic view of the pore walls in SBC4/Gel, from which one can see that the SBC nanofibers adhere to the surface of the pore walls, resulting in an increased specific surface area and roughness of the gelatin scaffold. The hydroxyl and sulfate groups on SBC can form hydrogen bonds with the amino groups in gelatin molecules, enhancing their interactions. Meanwhile, the high aspect ratio of SBC fibers makes them uniformly dispersed in the gelatin solution, allowing SBC to adhere better to gelatin. Taken together, the composite scaffolds have both micropores and a network of nanofibers, which is closer to the microstructure of the ECM supported by a network of collagen fibers and is therefore assumed to be more conducive to cell growth.

**Fig. 6.**
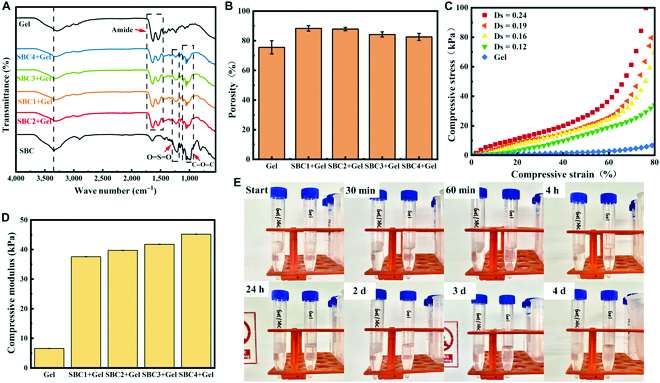
(A) FT-IR spectra, (B) porosity, (C) compression stress–strain curves, and (D) compressive modulus of Gel and SBC/Gels. (E) Photos showing the changes of appearances in physiological conditions within 4 d confirmed that SBC4/Gel degraded at a slower rate than gelatin.

Figure 6A compares the FT-IR spectra of the gelatin scaffold and the composites. The SBC/Gel composite scaffold contains characteristic bands for SBC, with absorption bands around 1,225, 1,080, and 890 cm^–1^ corresponding to the O=S=O asymmetric stretching vibration, the C–O stretching vibrations, and C–H deformation vibrations. The SBC/Gel composite scaffolds also have the characteristic bands of gelatin, namely, 1,631 and 1,532 cm^–1^ for amide I and amide II [27]. The SBC/Gel composites contain not only the characteristic bands of SBC but also those of gelatin, which proves that the composite scaffold is made of a combination both materials. More importantly, the sharp absorption band of the free hydroxyl group in SBCs appeared at 3,352 cm^–1^, while those of the SBC/Gel composite scaffold shifted to a lower wave number (3,338 cm^–1^) and had a blunt shape [28], indicating formation of hydrogen bonds between the gelatin and SBC molecules in the SBC/Gel composites, allowing tight binding of nanofibers to the gelatin.

By using a weighing method, we quantified the effect of sulfate substitution on the porosity of the composites and sole gelatin (Fig. 6B). All 4 SBC/Gel composites contain higher porosity (82.56% to 88.31%) than that of the gelatin material (75.57%), with gradual decrease from 88.31% to 82.56% as the degree of SBC substitution increased. It is possible that the SBC surface is rich in sulfate groups and hydroxyl groups, which create stronger interactions with gelatin and tighten the pore channels, resulting in a gradual decrease in porosity. High porosity facilitates the entry and exit of nutrients and metabolites and is therefore a prerequisite for tissue engineering scaffolds.

As the link between the cells and the tissue, the scaffolds are required to have certain compressive properties in order to withstand external pressure and maintain the integrity of the tissue. We therefore assessed the mechanical properties gelatin and SBC/Gel materials by the compression stress–strain curves shown in Fig. 6C. As the degree of sulfate substitution in the composite increases, the stress required for the same deformation gradually increases. By calculation of the compression modulus (Fig. 6D), gelatin has a compression modulus of 6.577 kPa, while SBC/Gel composites have increasing compression modulus of 3.759, 3.971, 4.178, and 4.518 kPa, respectively, as the degree of substitution increases. This indicates a gradual increase in the strength of the composites with better support capability, which is consistent with the improved stability (vide infra). This is probably because the interactions between the SBC cellulose crystals and the surface of the pore wall of the gelatin scaffold gradually increases and has a greater ability to improve the strength of the gelatin matrix.

In order to analyze the in vitro degradation of the material, gelatin scaffold and SBC4/Gel composite scaffold were incubated in PBS and observed for 4 d (Fig. 6E). The Gel aerogel showed significant degradation only after half an hour and completely dissolved in PBS after 1 d. In comparison, the SBC4/Gel aerogel still remained intact on the fourth day. It can be seen that the addition of SBC greatly improves the stability of the gelatin aerogel in PBS. This phenomenon is due to the addition of SBC nanofibers, which resulted in enhanced intermolecular hydrogen bonding and electrostatic interaction between the composites, resulting in a decrease in the degradation rate of the blended SBC/Gel composite scaffold. This result implies that the degradation rate of the scaffold material can be appropriately regulated by the content of BC nanofibers within the composite.

### Biocompatibility and stability of SBC/Gel composite scaffolds with different degrees of substitution

**Fig. 7.**
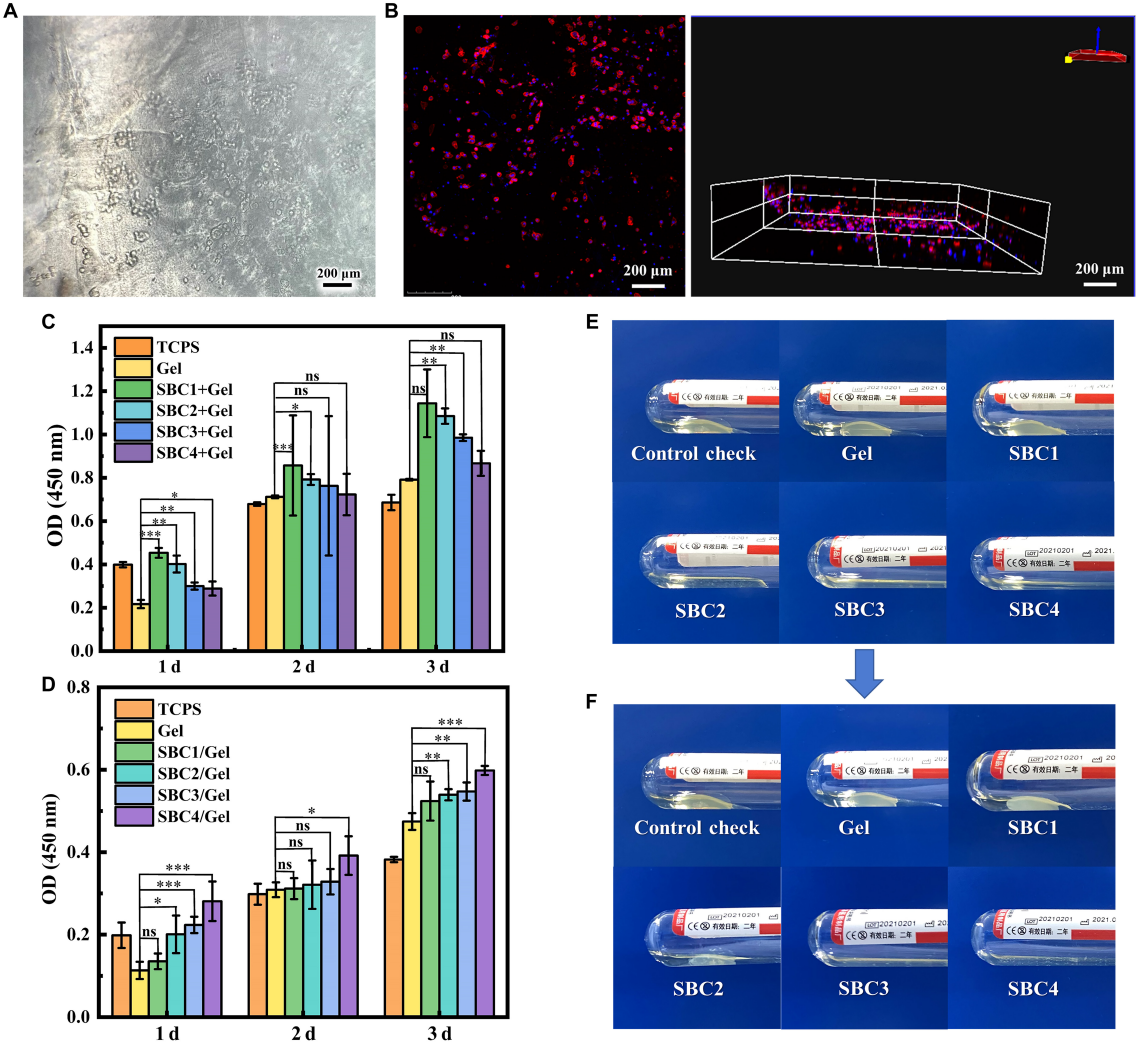
(A) Bright filed microscopic and (B) confocal microscopic images of L02 cells cultured on SBC4/Gel for 24 h. CCK-8 assay of (C) L02 cells (RRID:CVCL_6926) and (D) MIHA cells (RRID:CVCL_SA11) cultured on both Gel and SBC/Gels for different durations of time. (E) Significant blood clots were observed in the SBC1/Gel after 15 min and (F) in the SBC2/Gel after 30 min but not in SBC3/Gel or SBC/4 gel. *P < 0.05; **P < 0.01; ***P < 0.001; ns, not significant.

In order to analyze the biocompatibility of SBC/Gel composites, we implanted L02 cells (RRID:CVCL_6926) and MIHA cells (RRID:CVCL_SA11) on gelatin and SBC/Gel scaffolds for different durations of time and used CCK-8 assay to detect the cell proliferation. The relative growth rate values of all 4 composite scaffolds were above 100% and the cytotoxicity was 0, indicating that the SBC/Gel composite scaffold had no inhibitory effect on cell proliferation (Fig. 7C and D). The OD of the tissue culture polystyrene (TPCS) group was higher than those of the SBC/Gel materials at the beginning of the cell culture (day 1), but then the SBC/Gel groups rapidly surpassed the TPCS group, following the same trend as in Fig. 4G. The reason may be that the cells initially adhered more easily to the TPCS [29,30]. However, the proliferation rate was significantly higher than that of TPCS as the cells adapted to the 3D material, for the 3D material provided a better growth environment with interlocking and interpenetrating 3D pore structures, whereas on the 2D material, the cell growth space was gradually restricted.

It can be seen that the numbers of both cells in the SBC/Gel composites were significantly higher than the number of cells in the Gel scaffold or on TCPS after 3 d of incubation. It is possible that the addition of SBC increased the roughness and porosity of the gelatin scaffold, which was beneficial to the adhesion and migration of cells. In addition, the addition of hydrophilic and negatively charged sulfate groups to the BC nanofibers enhanced the adhesion between the cells and the material, which therefore enhanced supported cell growth. Although the average OD values of both cells had different trends as the substitution degree of SBC changes from 0.12 to 0.24, the cell activities were not significantly different after statistical analysis of the data. Nevertheless, both cell types grew better on the 3D materials than on the gelatin counterparts, confirming the effectiveness of the SBC in promoting cellular growth.

As SBC shares similar structure with heparin, a widely used anticoagulant, we also assessed whether the scaffold material has good hemocompatibility and anticoagulant effects. By APTT test, the time taken for plasma to clot when APTT reagent was added to the PPP with Ca^2+^, and we found that the APTT value for gelatin was 35.4 s, similar to that of blank control (43.2 s), which are within the normal range of reference values. In stark contrast, the APTT values for the SBC/Gel composites exceeded the upper limit of the instrument (120 s). To better compare between the 4 composite scaffolds, we extended the experiment time and recorded the appearances of clots formed in each group. The clots were evident in both the gelatin and blank control groups within 1 min, while it took 15 and 30 min for the clots to form in SBC1/Gel and SBC2/Gel composite groups at 30 min (Fig. 7E and F). It took even longer time to form clots in SBC3/Gel and SBC4/Gel composites, indicating better anticoagulant properties as the degree of sulfation substitution increased. Obviously, the anticoagulant properties of the SBC/Gel composites originate from the strongly negatively charged sulfate group, which enhances the affinity between antithrombin III and thrombin, causing rapid inactivation of thrombin and thus achieving the anticoagulant effect [31].

## Conclusion

We prepared SBC fibrous nanomaterials (20 to 50 nm in diameter and >20 μm in length) with an ultrahigh aspect ratio for building 3D scaffolds of hepatocyte culture. Simply by adjusting the reaction time, one can control the degree of sulfation, which would break the strong intermolecular forces improved the dispersion of BC fibers. After introducing a certain amount of sulfate ester groups, the SBC shows GAG-like properties and behaves more like the ECM with cytocompatibility. We then prepared 4 sets of SBC/Gel porous composites by homogenization and freeze-drying gelatin and SBC. The composites provide 3D micron macropores by gelatin and fibrous network nanostructures by SBC, which is very similar to natural ECM. SBC as a nanomaterial effectively solves the problem of poor mechanical properties of gelatin materials, while the SBC/Gel composite has better porosity and compression properties. The CCK-8 and APTT tests confirmed that the SBC/Gel material has good cytocompatibility and anticoagulation properties. Therefore, the composite material has a promising application as a scaffold material for cell culture and is expected to be used in drug screening and liver tissue engineering.

## Data Availability

The data that support the findings of this study are available from the corresponding author.
